# Jellyfish Search-Optimized Deep Learning for Compressive Strength Prediction in Images of Ready-Mixed Concrete

**DOI:** 10.1155/2022/9541115

**Published:** 2022-08-01

**Authors:** Jui-Sheng Chou, Stela Tjandrakusuma, Chi-Yun Liu

**Affiliations:** National Taiwan University of Science and Technology, Taipei, Taiwan

## Abstract

Most building structures that are built today are built from concrete, owing to its various favorable properties. Compressive strength is one of the mechanical properties of concrete that is directly related to the safety of the structures. Therefore, predicting the compressive strength can facilitate the early planning of material quality management. A series of deep learning (DL) models that suit computer vision tasks, namely the convolutional neural networks (CNNs), are used to predict the compressive strength of ready-mixed concrete. To demonstrate the efficacy of computer vision-based prediction, its effectiveness using imaging numerical data was compared with that of the deep neural networks (DNNs) technique that uses conventional numerical data. Various DL prediction models were compared and the best ones were identified with the relevant concrete datasets. The best DL models were then optimized by fine-tuning their hyperparameters using a newly developed bio-inspired metaheuristic algorithm, called jellyfish search optimizer, to enhance the accuracy and reliability. Analytical experiments indicate that the computer vision-based CNNs outperform the numerical data-based DNNs in all evaluation metrics except the training time. Thus, the bio-inspired optimization of computer vision-based convolutional neural networks is potentially a promising approach to predict the compressive strength of ready-mixed concrete.

## 1. Introduction

Structures like buildings, bridges, highways, and dams are currently built using concrete as their construction material, owing to its numerous advantages, such as strength, durability, and versatility. Its compression capacity, adaptability, and resistance to climate-induced erosion and corrosion make concrete one of the best construction materials. Compressive strength is one of the principal mechanical properties of concrete that is directly related to the safety of the structures that are built from it. The compressive strength of concrete must comply with relevant standard codes, which vary among countries.

To determine the compressive strength of concrete, a cubic or cylindrical sample is typically tested using a compressive testing machine after the required curing time. These tests are labor-intensive and time-consuming. Methods such as regression methods and numerical simulation have been proposed to solve this problem and to predict the compressive strength of concrete. However, the complex nonlinear correlation between relevant variables makes the obtaining of accurate values of compressive strength very difficult.

With the advances of artificial intelligence (AI) and increases in computing power [1, 2], deep learning (DL) is being applied in an increasing number of fields. DL, which is a form of AI, has been shown to be effective in making more accurate predictions than conventional methods in many situations. One DL technique, computer vision, is often used to extract information from visual media, such as images and videos. Used in various fields, computer vision-based technique is effective for image classification, object detection, and semantic segmentation.

Several studies of the prediction of concrete compressive strength have involved the use of DL techniques [3] to improve model performance, but few have involved image recognition. The latest study of the use of image recognition to determine compressive strength had good results, but it used only 74 sets of concrete data and a single-layer convolutional neural network [4]. To examine and improve the effectiveness of image recognition, in this study, a large dataset of ready-mixed concrete is used with convolutional neural networks (CNNs) that involve a prediction model with deep layers to extract high-level features from inputs.

Model accuracy is often evaluated with the use of a cross-fold validation or random split method to partition the source data for the testing of the training model [5]. Such methods are often called into question as overfitting occurs owing to the information leakage within the original dataset in the training process. Therefore, when putting the model into practice, it often shows a relatively poor forecast performance. Because the concrete data are accumulated over time in ready-mixed plants, the built model should be tested via a latest dataset to reflect its reasonable prediction accuracy in future use.

In this investigation, the effectiveness of the computer vision-based approach in predicting the compressive strength of ready-mixed concrete by converting numerical data to images is tested. In much research, the prediction of the compressive strength of concrete uses numerical data as inputs. The results thus obtained using those computer vision-based techniques are compared with those obtained using numerical data. With this logic, a collection of numerical values that are represented as images are the inputs to a DL technique that uses CNN-based models, which have been shown to provide accurate image classification in the domain of computer vision.

The effectiveness of the computer vision-based technique was tested by comparing the results with those of another DL technique that uses deep neural networks (DNNs) with numerical data for model construction. To maximize the accuracy, a metaheuristic optimization algorithm was used to finetune the hyperparameters of the best DL models. Instead of using the cross-fold validation or random split method within the original dataset, a newly collected dataset in the upcoming year was used for the testing of the training model. This approach meets the practical needs and operations in estimating compressive strength at ready-mixed concrete plants.

This paper is organized as follows.  Section 2 reviews the relevant literature.  Section 3 describes the methodology and performance metrics that are used herein.  Section 4 presents the collection and preprocessing of data, implementation of the DL models, the experimental results obtained using the optimized DL models, and sensitivity analysis of modeling performance. The final section summarizes the findings and limitations of the method and makes recommendations for future studies.

## 2. Literature Review

### 2.1. Conventional Compressive Strength Prediction of Ready-Mixed Concrete

**Figure 1:**
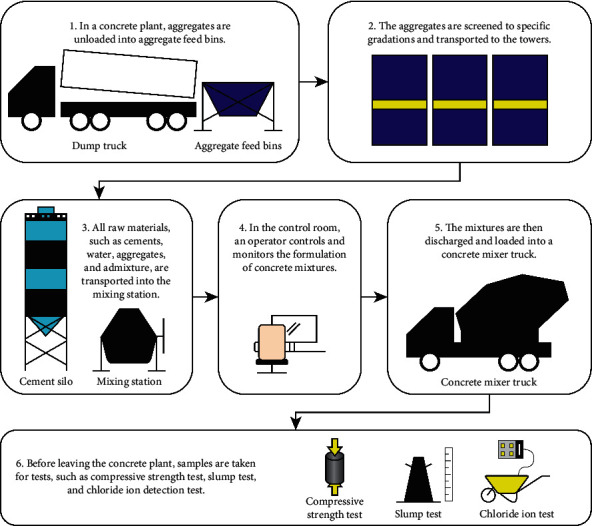
Ready-mixed concrete manufacturing process.

Ready-mixed concrete is typically manufactured in a concrete plant before being transported to a construction site. In a concrete plant, ready-mixed concrete is manufactured by combining several raw materials with a specific design mix ratio to create concrete with certain desirable properties.  Figure 1 presents the manufacturing process of ready-mixed concrete.

The compressive strength of concrete is commonly tested using a compression test machine, which performs a mechanical test to measure the maximum compressive load that can be borne by a concrete sample [6]. Before testing, the sample must be cured for a specified curing period. Non-destructive tests (such as ultrasonic or pulse velocity tests [7] and conductivity tests [8]) have also been proposed to determine the compressive strength due to the lack of correlation between the standard compression test value with the real strength of concrete in a structure. These tests, however, have disadvantages with respect to time, cost, and labor.

Owing to the disadvantages of mechanical tests, empirical models [9, 10] for calculating the compressive strength of concrete have been developed. Empirical methods (e.g., multiple linear regression), however, have been shown to be somewhat ineffective for calculating the compressive strength of concrete because of the nonlinear behavior in relevant concrete variables. The compressive strength of concrete is influenced by numerous factors, as it is formed by complex reactions among concrete materials (such as cement and aggregates) and the environment (as in curing) [11].

### 2.2. Deep Learning to Determine Concrete Compressive Strength

In recent years, the field of artificial intelligence (AI) has grown very rapidly. AI methods are used in a wide variety of fields, including seismology [12], energy systems [13], and civil engineering [14]. In several studies, AI has been used to determine the concrete compressive strength, using real data for concrete to build a prediction model. During the training of the prediction model, various composite materials of concrete, such as cement, water, sand, and gravel, are used as predictors to yield a model that best fits the given training data. After validation, the model is then used to predict the compressive strength.

An advanced branch of AI, deep learning (DL), has performed excellently in fields such as computer vision [15]. Many studies [16–18] have shown that DL exhibits outstanding prediction performance, especially in image and video recognition. In this field, the commonly used DL techniques include those based on convolutional neural networks (CNNs) [19]. A recent study confirmed that the CNN model (Visual Geometry Group, VGG) achieved a 98% accuracy in concrete compressive strength prediction, which was 2% and 12% greater than the machine learning models, random forest (RF) and support vector regression (SVR), respectively [20].

### 2.3. Hyperparameter Optimization with Metaheuristic Algorithm

In the training of the DL models, additional optimizers are often required, as the models have several hyperparameters (such as the epsilon of batch normalization, batch size, epoch, learning rate, and dropout rate) that influence their predictive performance [21, 22]. To find the values of hyperparameters that yield the best prediction model, optimization algorithms (such as the greedy algorithm [23]) that are based on iterative methods (such as gradient descent [24]) or heuristic methods [25] are often used. However, such methods may not always lead to the optimal solution and consume a significant computational time compared to modern metaheuristic algorithms.

The metaheuristic algorithm, with its ease of implementation and effectiveness in various fields, is becoming increasingly popular for use in solving optimization problems. Recently, several newly developed metaheuristic optimizers have outperformed the well-known metaheuristic algorithms [26, 27]. The Jellyfish Search (JS) algorithm [27], in particular, has great efficacy because it requires little tuning of algorithm-specific parameters. Consequently, the JS algorithm was used in this study to optimize the DL models.

## 3. Methodology

### 3.1. Deep Learning and Computer Vision-Based Techniques

#### 3.1.1. Deep Neural Networks

**Figure 2:**
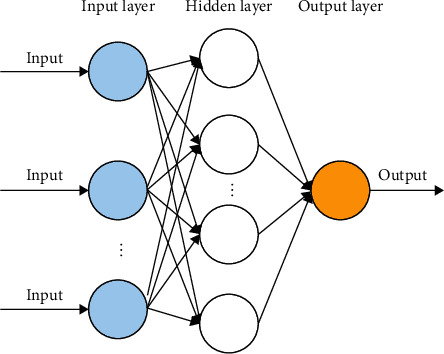
Simple ANN model architecture.

Artificial neural networks (ANNs) consist of information processing units that are arranged in layers similar to neurons in the human brain. An ANN typically comprises layers of three types: an input layer, hidden layers, and an output layer. The architecture that is used in a deep learning model typically consists of more than four hidden layers.  Figure 2 displays a simple ANN model architecture.

An input layer receives data and an output layer generates a prediction. In the hidden layers, inputs are processed and the information that is obtained from the processes is passed to the next layer. Values from the input layer are transformed by multiplying them by weights and adding bias values.

Several types of ANN vary in implementation. A fully connected neural network is an ANN that consists of connected neurons. In such an ANN, all neurons in a layer are connected to the neurons in the next layer. Likewise, standard feedforward neural networks (FNNs) consist of numerous connected neurons, and each connection transmits information to other neurons in one forward direction [28].

Notably, internal hyperparameters affect the learning of an ANN model. A hyperparameter is a constant parameter that is set before the training begins. Some examples of hyperparameters in ANNs are the number of hidden layers, learning rate, batch size, and epoch. In contrast, parameters such as weights and bias values change throughout the learning process.

**Figure 3:**
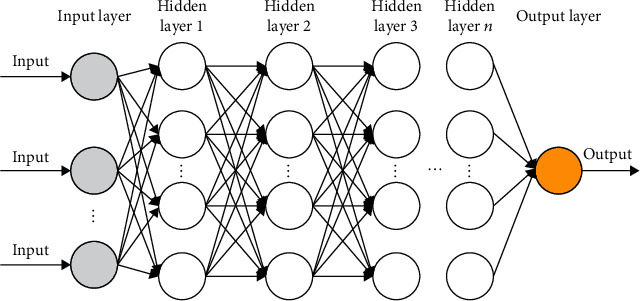
Deep neural network (DNN) model architecture.

A deep neural network (DNN) is a neural network that differs from a typical ANN with respect to architecture. DNNs have multiple hidden layers (Figure 3) that are used to extract high-level features from the input data. Additional layers typically correspond to additional parameters (such as weights and biases) in a model. Accordingly, DNNs can capture complex nonlinear relationships [28].

#### 3.1.2. Convolutional Neural Network-Based Models

A convolutional neural network (CNN or ConvNet) is a connected neural network that is generally effective for solving computer vision problems, such as image feature extraction, classification, object detection, and semantic segmentation. A CNN commonly learns patterns by processing image or video data. It can detect objects, identify the locations of the objects, and differentiate or segment them inside an image.

**Figure 4:**
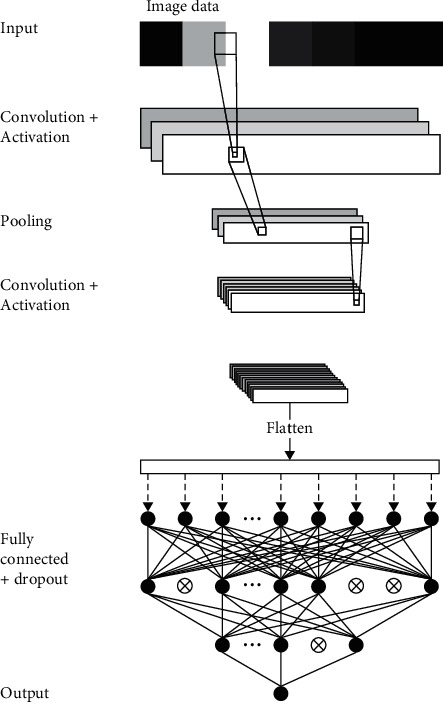
Generic convolutional neural network (CNN) model architecture.

A generic CNN usually comprises an input layer, multiple hidden layers, which include convolutional layers, pooling layers, fully connected layers, and dropout layers, and an output layer (Figure 4). In the input layer, the model receives images as inputs and creates input tensors that contain the pixel values of the images. Input matrixes of dimensions *w* × *h* × *c* are then fed to the hidden layer, where *w* represents the width of the image, *h* represents the height of the image, and *c* represents the number of channels. A standard colored image typically has three channels for red, green, and blue.

The convolutional layer in the CNN model processes the previous matrixes into smaller forms without losing any feature by generating weight values of a filter or kernel of a certain size (*m* × *m*) and then multiplying the filter (*n* × *n*) by the input matrixes. Convolution operation is defined as follows [29]:

(1) $$\begin{array}{c} C=I⊗F. \end{array}$$

**Figure 5:**
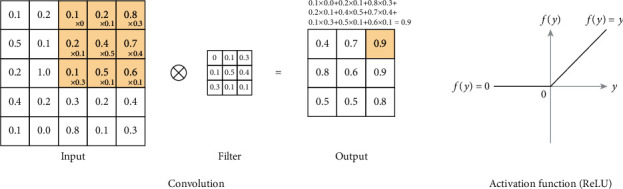
Convolutional layer multiplication process and plot of ReLU.

Here, *I* is the input image data; *F* is the filter; ⊗ denotes the convolution operation; *C* is the convolution map of size (*o* × *o*), in which *o* =  *m* − *n*+2*zp*/*s* + 1; *s* is the stride and denotes the number of pixels by which *F* is sliding over *I*; and zp is the zero padding. Usually, it is necessary to add a bounding of zeros around *I* to preserve complete image information. The values thus obtained are summed (Figure 5). Sliding over all parts of input matrixes, the convolutional layer generates, as an output, a new feature map of certain features in the image.

After the multiplication processes, a CNN model typically applies an activation function that introduces nonlinearity to the model to help it learn complex patterns in the data. A general form of activation function is defined as follows:

(2) $$\begin{array}{c} C_{m}=f\left(C\right). \end{array}$$

*C*_*m*_ is the convolution map after applying the nonlinear activation function *f*. Of the many available activation functions, the rectified linear unit (ReLU) is commonly used, as it provides better training results than other activation functions [30]. A ReLU function is a simple calculation that returns the original input values or sets the value to zero if the input is less than or equal to zero (Figure 5).

**Figure 6:**
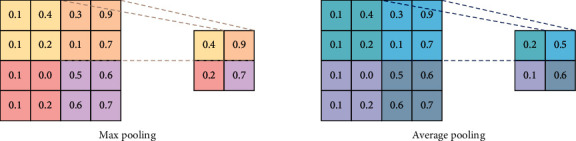
Example of max pooling and average pooling.

The pooling layer in the model reduces the size of the input matrixes by reducing the number of parameters and the amount of computation in the network, preventing overfitting. Similar to a convolutional layer, a pooling layer takes several input values inside a filter from the previous layer and the filter is shifted over some pixels at a time until all parts of the input matrix are processed. Common pooling layer types are average pooling or max pooling (Figure 6). The pooling operation also called downsampling operation is expressed as follows:

(3) $$\begin{array}{c} P_{m}=P_{o}\left(C_{m}\right), \end{array}$$

where *P*_*m*_ is the pooling map and *P*_*o*_ is the pooling operation.

After the operation of several convolutional layers and pooling layers, a CNN model typically flattens the output matrix of the previous layer into a single vector of values. The single vector of values is input to a fully connected layer to extract the features that were learned in the previous layers and to classify the input images. In this layer, the probabilities that an object in the input image is a member of the possible classes are calculated. The model output of the i^th^ fully connected hidden layer is expressed as follows [29]:

(4) $$\begin{array}{c} Y^{i}=f\left(H^{i}\right), \end{array}$$

where the weight sum vector *H*^*i*^ is

(5) $$\begin{array}{c} H^{i}=w^{i}Y^{i-1}+B^{i}. \end{array}$$

*w* is the connected weight of the artificial neurons. *f* is a nonlinear activation function (e.g., sigmoid, Tanh, ReLU, etc.). The bias value *B*^*i*^ defines the activation level of the artificial neurons.

In neural networks, when the parameters of a layer change, so do the distribution of inputs to subsequent layers. These shifts in input distributions can be problematic for neural networks. To alleviate this concern, many normalization operations, such as Batch Normalization (BN), Layer Normalization (LN), and Instance Normalization (IN), have been proposed. For example, given an input batch of height *h* and width *w* with *n* samples and *c* channels *x* ∈ *R*^*n*×*c*×*h*×*w*^ , BN normalizes the mean and standard deviation for each individual feature channel during training [31].

(6) $$\begin{array}{c} BN\left(x\right)=\gamma \left(\frac{x-\mu_{B}}{\sigma_{B}}\right)+\beta , \end{array}$$

where *γ*, *β* ∈ *R*^*c*^ are referred to as the scale and the shift parameters for the channel; *μ*_*B*_, *σ*_*B*_ ∈ *R*^*c*^ are the mean and standard deviation, respectively, computed across batch size and spatial dimensions independently for each feature channel.

Adding a dropout layer is an effective regularization technique to improve the generalization capability and mitigate overfitting of models. Dropout function can be formulated as follows [32]:

(7) $$\begin{array}{c} {\tilde{f}}^{l}\left(x_{i}\right)=f^{l}\left(x_{i}\right)-m_{i}^{l}*f^{l}\left(x_{i}\right), \end{array}$$

where ∗ denotes the element-wise product and *f*^*l*^(*x*_*i*_) and ${\tilde{f}}^{l}\left(x_{i}\right)$ are the original feature and distorted features, respectively. In addition, ∈ *m*_*i*_^*l*^{0,1}^*d*^*l*^^ is the binary mask applied on feature map *f*^*l*^(*x*_*i*_) in which *d*^*l*^ is the dimension of the feature map of *l*-th layer, and each element in *m*_*i*_^*l*^ is drawn from Bernoulli distribution and set to 1 with the dropping probability. Undoubtedly, implementing dropout on the features in the training phase will force the given network paying more attention on those non-zero regions, and partially solve the overfitting.

**Figure 7:**
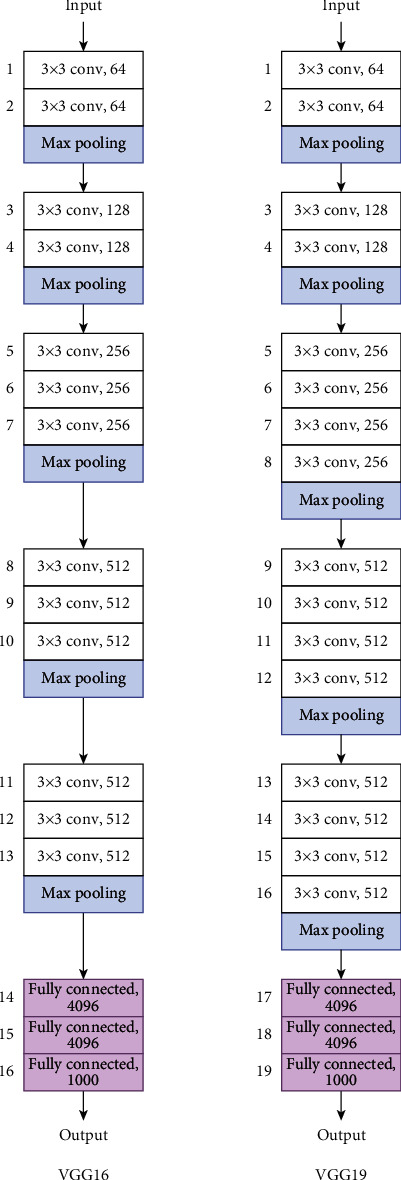
VGG16 and VGG19 models' architectures.

In this decade, various CNN models and their advanced variants have been developed. Some common and popular CNN models are VGG [33], residual neural networks (ResNets) [34, 35], Inception [36, 37], extreme inception (Xception) [38], MobileNet [39, 40], DenseNet [41], NASNet [42], and EfficientNet [43]. These CNN-oriented models have different architectures, which are briefly introduced as follows:

*(1) VGG*. VGG [33] uses a very small kernel (3 × 3) rather than one of a previously common size, 5 × 5 or 7 × 7, which would have a wider scanning area. The small kernel is used uniformly throughout all layers. Although the overall architecture is simple, the VGG has an enormous number of parameters.  Figure 7 displays the architectures of two common VGG models, VGG16 and VGG19, which comprise 16 and 19 deep layers, respectively. In the figure, the convolutional layer is denoted as “<kernel size> Conv, <filter>.”

*(2) ResNet*. Increasing the depth of a CNN by adding layers to its architecture up to a certain limit should help the corresponding CNN model to learn more complex features, but a vanishing gradient problem typically prevents the effective training of a CNN model in many-layered networks. A vanishing gradient problem can prevent the weights in the network from being updated, potentially stopping the training of the CNN model. To solve this problem in residual neural networks (ResNets), the network implements “residual connections” or “skip connections.”

**Figure 8:**
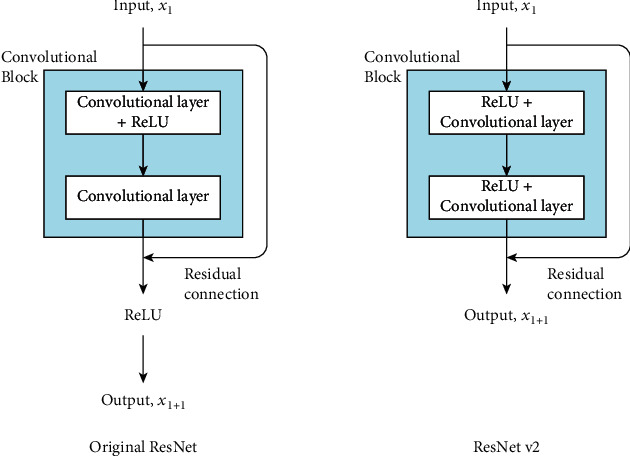
Residual connection.

**Figure 9:**
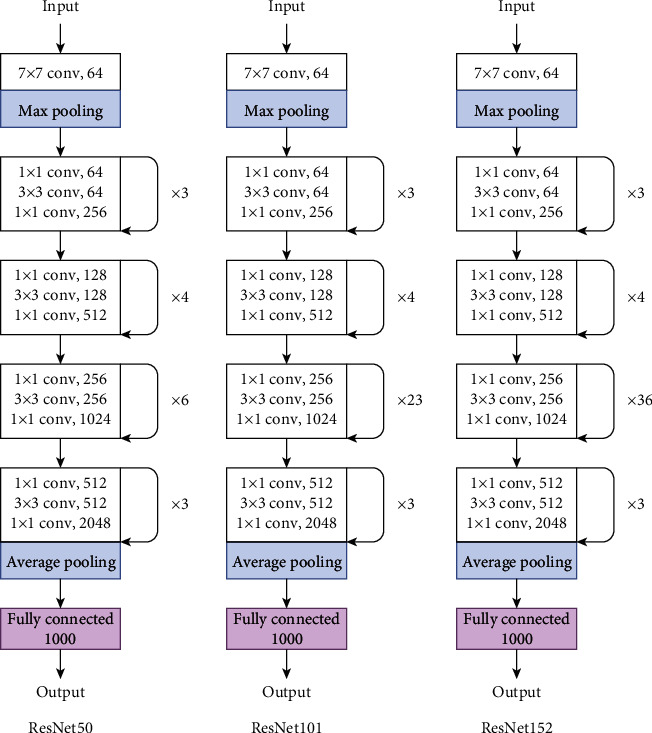
ResNet model architecture.

A residual connection refers to a shortcut connection that is added inside a CNN architecture to allow information to be passed or added through layers of the convolutional block (Figure 8). In the original ResNet, a shortcut connection is added before the activation function is implemented, while in ResNet v2 [34], activation functions are implemented before the convolutional layer and the shortcut connection is added after.  Figure 9 presents the architectures of ResNet50, ResNet101, and ResNet152, which comprise 50, 101, and 152 deep layers, respectively.

*(3) Inception*. Inception architecture [36] is the first CNN model architecture that exhibits the advantages of branching a convolutional path into multiple paths. In Inception, the CNN model uses filters of various sizes in various paths. At the end of the block, the model concatenates the outputs of the paths. In Inception-v3 [36], the Inception model is improved by changing the original 5 × 5 and 7 × 7 convolution kernels to two 3 × 3 and three 3 × 3 convolutional kernels, respectively. These changes in the architecture help the model reduce the amount of computation that is required during the training process.

**Figure 10:**
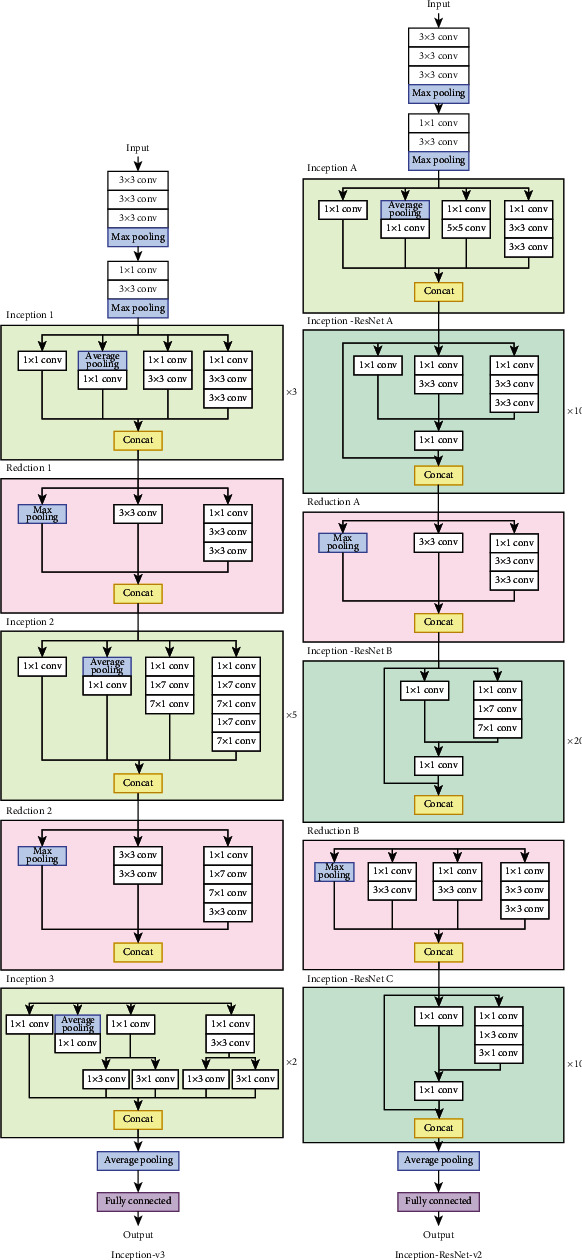
Inception-v3 and Inception-ResNet-v2 models' architectures.

In Inception-ResNet-v1 [37] and Inception-ResNet-v2 [37], the original inception blocks are converted into residual inception blocks. The Inception-ResNet-v2 model differs from the Inception-ResNet-v1 model in that it is more computationally burdensome. However, it outperforms the original Inception and ResNet models.  Figure 10 displays the Inception-v3 and Inception-ResNet-v2 models' architectures.

**Figure 11:**
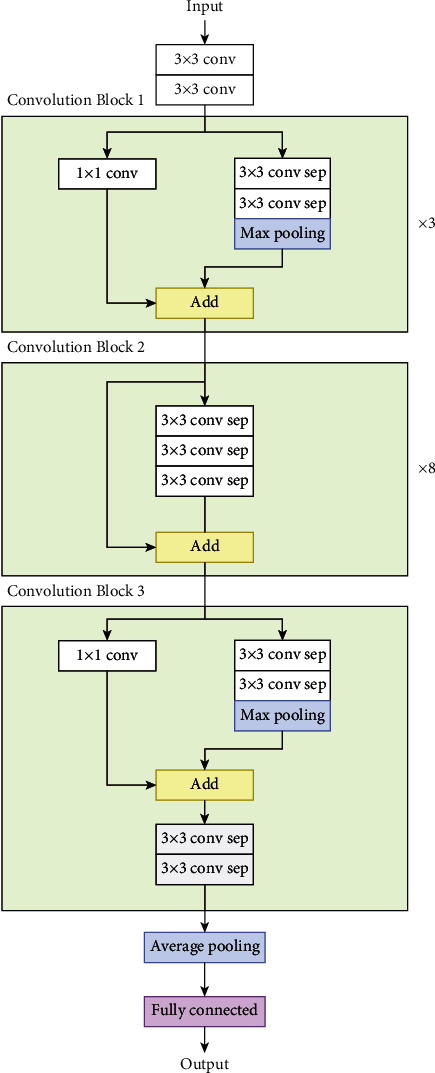
Xception model architecture.

*(4) Xception*. The Xception (or Extreme Inception) [38] architecture (Figure 11) is inspired by the Inception model. In Xception, the original inception blocks are replaced by depthwise separable convolutions. A depthwise separable convolution consists of a depthwise convolution and a 1 × 1 convolution. A depthwise convolution is a spatial convolution that performs convolutional multiplications independently over each channel. In depthwise convolution, a convolutional kernel only iterates one channel of the input, not all channels.

*(5) MobileNets*. MobileNets [39] refer to a type of CNN model whose objectives are to reduce the number of parameters and the number of computations while maintaining accuracy. Accordingly, MobileNets use depthwise separable convolutions. They are typically used in mobile devices or embedded applications, and so have a small architecture. In MobileNets, width multiplier and resolution multiplier hyperparameters are implemented to thin the network and to rescale the input image, respectively.

**Figure 12:**
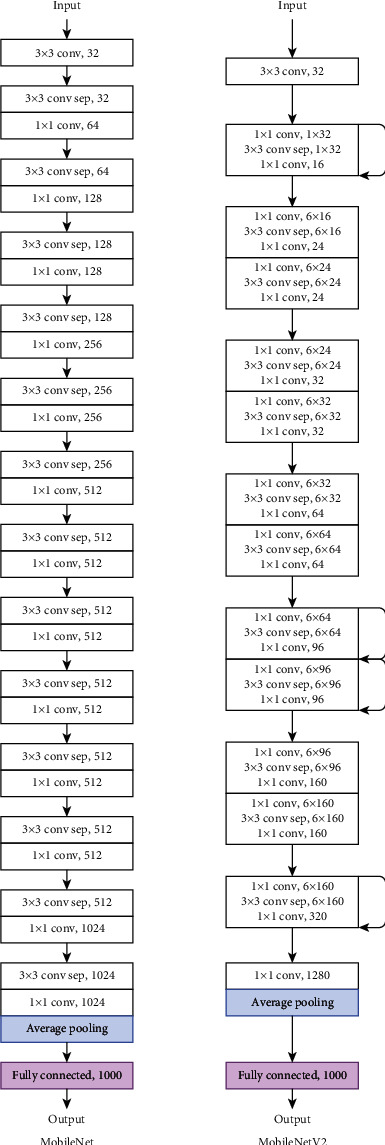
MobileNet and MobileNetV2 models' architectures.

Similar to the original MobileNet, MobileNetV2 [40] is built for mobile devices. In MobileNetV2, an inverted residual structure, which consists of linear bottleneck layers, is used. An inverted residual structure expands a low-dimensional feature map to a high-dimensional one, uses depthwise convolutions, and projects back features to a low-dimensional representation using a linear convolution. MobileNetV2 has fewer parameters than the original MobileNet.  Figure 12 displays the original MobileNet and MobileNetV2 architectures.

**Figure 13:**
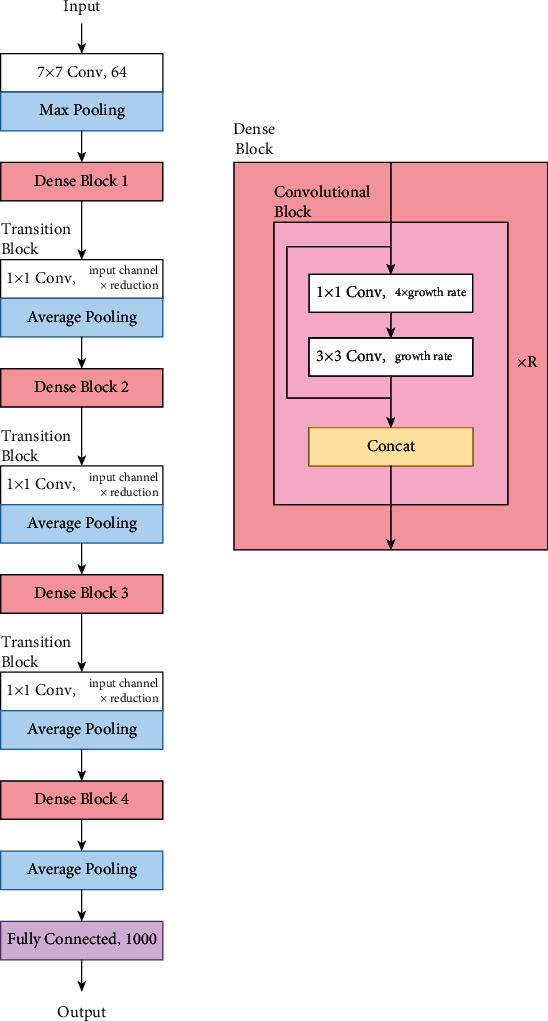
DenseNet model architecture.

*(6) DenseNet*. The main intent of a dense convolutional network (DenseNet) [41] is to use short connections between layers by connecting the network layers to every other layer in the forward direction. Therefore, the inputs of each network layer include the feature maps of all preceding layers. This approach has been shown to improve the accuracy of a CNN.  Figure 13 displays the DenseNet architecture.

**Figure 14:**
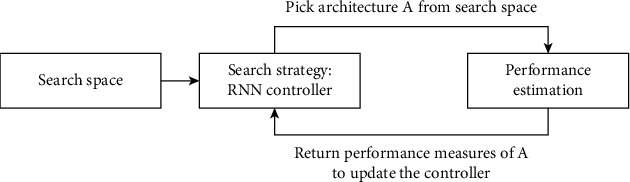
Neural architecture search method.

**Figure 15:**
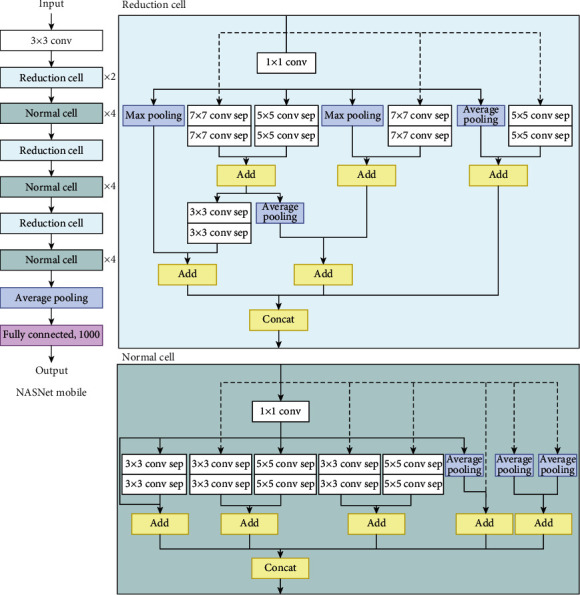
NASNet-A mobile model architecture.

*(7) NASNet*. The neural architecture search network (NASNet) [42] is used to solve the problem of finding a good CNN architecture by finding a neural network architecture or the best combination of parameters in a CNN with a recurrent neural network (RNN) acting as a controller.  Figure 14 presents the neural architecture search method that is used in a NASNet model.  Figure 15 displays one of the model architectures, NASNet-A, for the mobile version, which is found using the neural architecture search method.

**Figure 16:**
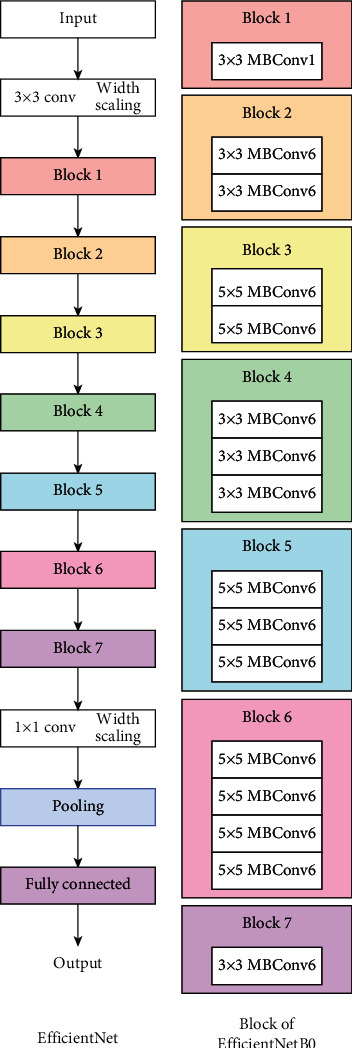
Efficientnet model architecture.

*(8) EfficientNet*. EfficientNet is a type of CNN model that uniformly scales all depth, width, and resolution dimensions using a compound scaling coefficient. A total of eight CNN models are developed based on this idea. The models are named EfficientNets followed by B0, B1, B2, B3, B4, B5, B6, and B7. The EfficientNet architecture includes a total of seven network blocks (Figure 16). The number of subblocks inside varies with the EfficientNet models that are used [43].

### 3.2. Metaheuristic Optimization Algorithm: Jellyfish Search Optimizer

One of the challenges that is associated with the deep learning models is the finding of optimal hyperparameters. To solve this hyperparameter optimization problem, a metaheuristic optimization algorithm is frequently used. Considerable research has been done on the development of metaheuristic algorithms, and some of them have become well known for their effectiveness in solving optimization problems [44–46]. The metaheuristic algorithms primarily vary in the balance between their two main phases—exploration and exploitation [47].

A newly developed metaheuristic optimization algorithm, the Jellyfish Search (JS) optimizer [27], has considerably outperformed many other well-known metaheuristic optimization algorithms and it requires less algorithm-specific parameter tuning than some well-known metaheuristic algorithms. The optimizer requires the setting of only two controlling parameters, which are the number of iterations and population size. In a JS optimizer, the population of jellyfish is initialized using a logistic map, which generates varying initial populations.

**Figure 17:**
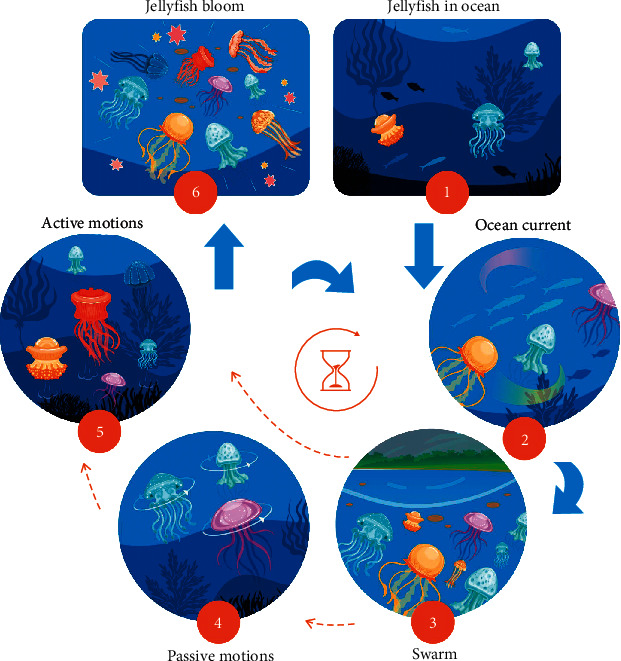
Phases of the jellyfish search algorithm.

Since the optimization algorithm is inspired by the behavior of jellyfish as they search for food in the ocean, the objective function of the JS optimizer is the location of jellyfish where it has the most food. In a JS optimizer, the exploration phase involves the movement of jellyfish as they follow ocean currents in search of food, while the exploitation phase involves the passive and active motions of the jellyfish inside a jellyfish swarm.  Figure 17 presents the six phases of jellyfish in the ocean [27], including phase 1: jellyfish in the ocean; phase 2: following the ocean current; phases 3–5: passive and active motions inside the jellyfish swarm that are switched to each other according to a time control mechanism; and phase 6: reach the jellyfish bloom.

#### 3.2.1. Movement Following Ocean Current

Ocean currents carry a large amount of food, attracting jellyfish to them, and thus jellyfish follow them. The following equation represents the direction of the ocean current, ($\overset{⟶}{trend}$), and the new location of a jellyfish after it moves, *X*_*i*_(*t* + 1) [27].

(8) $$\begin{array}{c} \overset{⟶}{\text{trend}}=X^{*}-3\times \text{rand}\left(0,1\right)\times \mu ,\\ X_{i}\left(t+1\right)=X_{i}\left(t\right)+\text{rand}\left(0,1\right)\times \overset{⟶}{\text{trend}}. \end{array}$$

Here, *X*^*∗*^ is the jellyfish at the best location, *μ* is the average location of all jellyfish, *X*_*i*_(*t*) are the current locations of the jellyfish at time *t*, and *X*_*i*_(*t* + 1) are the updated locations of the jellyfish at time (*t*+1).

#### 3.2.2. Motions Inside Jellyfish Swarm

The motions of jellyfish in a swarm can be grouped into passive motion (type A) and active motion (type B). Passive motion signifies a movement of a jellyfish around its original position, and active motion signifies its movement to another position. Initially, most jellyfish exhibit type A motion, but after some time, more jellyfish exhibit type B motion [27]. The new location of a jellyfish that exhibits A motion is formulated as follows:

(9) $$\begin{array}{c} X_{i}\left(t+1\right)=X_{i}\left(t\right)+0.1\times \text{rand}\left(0,1\right)\times \left(Ub-Lb\right), \end{array}$$

where rand(0,1) is a random number between 0 and 1, *Ub* is the upper bound on the search space, and *Lb* is the lower bound on the search space.

For type B motion, one other jellyfish, *X*_*j*_, is randomly selected for use in determining the new location of the jellyfish of interest, *X*_*i*_. If the amount of food at the location of *X*_*j*_ exceeds that at the location of *X*_*i*_, then *X*_*i*_ will move toward *X*_*j*_. Otherwise, *X*_*i*_ will move away from *X*_*j*_. The direction of type B motion $\left(\overset{⟶}{\text{Direction}}\right)$ and the updated jellyfish location are given by the following equations for minimization problems:

(10) $$\begin{array}{c} \overset{⟶}{\text{Direction}}=\left\{\begin{array}{c} X_{j}\left(t\right)-X_{i}\left(t\right),\;\text{if }f\left(X_{i}\right)\geq f\left(X_{j}\right),\\ X_{i}\left(t\right)-X_{j}\left(t\right),\;\text{if }f\left(X_{i}\right)<f\left(X_{j}\right), \end{array}\right.\\ X_{i}\left(t+1\right)=X_{i}\left(t\right)+\text{rand}\left(\text{0,}1\right)\times \overset{⟶}{\text{Direction}}, \end{array}$$

where *f*(*X*_*i*_) and *f*(*X*_*j*_) denote the objective functions at locations *X*_*i*_ and *X*_*j*_, respectively.

#### 3.2.3. Time Control Mechanism

A time control mechanism in a JS optimizer determines the type of jellyfish motion and controls the switching between the phases of the JS optimizer (following an ocean current and moving inside a jellyfish swarm). The equation below provides the time control function, *c*(*t*).

(11) $$\begin{array}{c} c\left(t\right)=\left|\left(1-\frac{t}{Max_{iter}}\right)\times \left(2\times \text{rand}\left(0,1\right)-1\right)\right|. \end{array}$$

Here, *t* is the time specified as the iteration number and *Max*_*iter*_ is the maximum number of iterations.

If the value of *c*(*t*) exceeds 0.5, then the jellyfish will follow the ocean current; if it is less than or equal to 0.5, the jellyfish will move in a jellyfish swarm [27]. To determine the type of jellyfish motion inside a jellyfish swarm (passive motion and active motion), the function 1 − *c*(*t*) is used. When rand(0,1) exceeds (1 − *c*(*t*)), the jellyfish will exhibit passive motion (type A). When rand(0,1) is less than (1 − *c*(*t*)), the jellyfish will exhibit active motion (type B). As *t* increases, the value of 1 − *c*(*t*) also increases [27].

#### 3.2.4. Algorithmic Flowchart and Pseudocode

**Figure 18:**
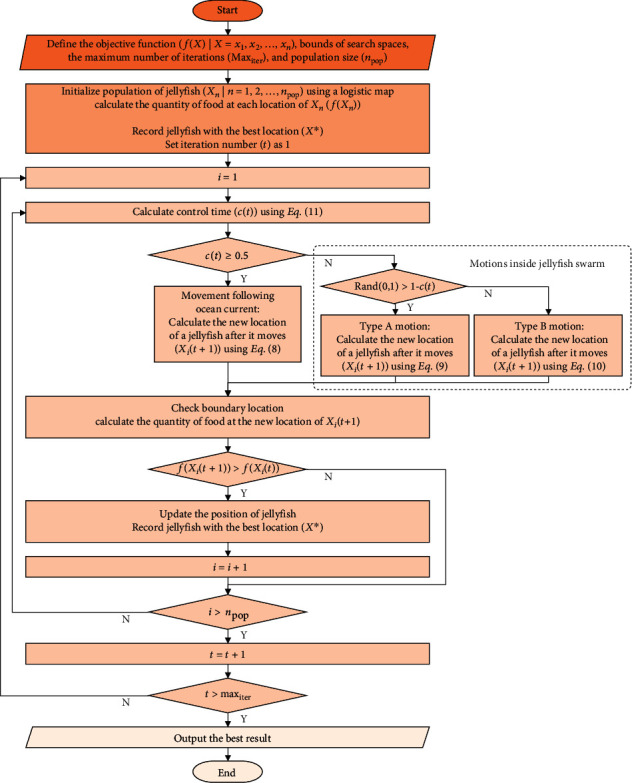
Algorithmic flowchart of the jellyfish search algorithm.

**Figure 19:**
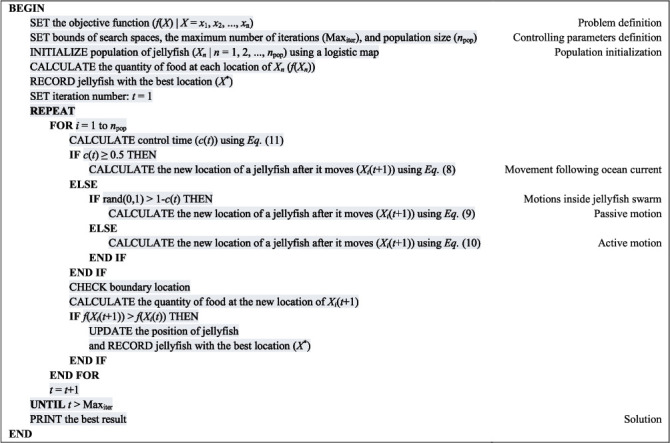
Pseudocode of the jellyfish search algorithm.

The algorithmic flowchart and the pseudocode of the JS algorithm, starting from problem definition, controlling parameters' definition, initialization, to the loop of phases, are presented in Figures 18 and 19, respectively.

### 3.3. Validation and Performance Evaluation

Validating the capability of the DL model that classifies data or analyzes datasets to predict a new dataset is essential. In neural network models, a loss function usually refers to the minimization of the prediction error. The training error, which is the average loss of the training sample, is not useful for evaluating the performance of the model because a low training error may indicate that the model is overfitting the training data, and so will generally perform poorly given new data [48]. The validation, therefore, should be conducted using a separate sample error.

During the development of a DL model, a dataset is typically split into three sets–the training set, the validation set, and the test set. The training set is used to learn the pattern of the inputs that correspond to a certain output; the validation set is used to evaluate the prediction error of the training model and to tune its hyperparameters; the test set is used to assess the error of the final model. No exact rule for splitting the dataset exists, as the split depends on the number and complexity of the available data.

#### 3.3.1. Validation Method

A validation set is used as the input of a previously trained prediction model to evaluate the performance of the model when used with new, never-seen-before data. The validation process is repeated multiple times with various hyperparameter combinations, and thus the purpose of using a validation set is to assess the performance of the training model and to find the optimal hyperparameters.

Two of the most popular methods for evaluating the generalization ability of the prediction model are holdout method and cross-validation. The holdout method randomly splits the data into a training set, a validation set, and a test set. The cross-validation method partitions a dataset into several subsets, implements the learning process on all but one of those subsets, and evaluates the performance using the left-out subset in turn. The cross-validation method is particularly suitable for a small dataset to enhance model validity.

For practical use in the ready-mixed concrete plant, the model is built based on the accumulated historical data, and subsequently will be used for a new concrete dataset in the prediction of compressive strength. To fairly reflect the prediction accuracy on-site, this study adapted the holdout method by training/validating the model with the whole historical dataset and testing it with newly collected concrete data in the upcoming year. By doing so, one would not overestimate the model performance in practice and could prevent information leakage from model training.

#### 3.3.2. Performance Metrics

The performance metrics that are used in this study are the mean absolute error (MAE), root mean square error (RMSE), mean absolute percentage error (MAPE), training time, and synthesis index (SI). The MAE is the average of the absolute differences between the actual and predicted values. Taking the absolute difference makes all error values positive, avoiding the false determination of an accurate prediction when negative and positive differences are summed.

Mean squared error (MSE) is the average of the squared differences between the actual and predicted values. The square root of the MSE, called the RMSE, is taken to the lower order of the MSE. MAPE is the average of the absolute errors divided by the actual values. The training time of various modeling techniques is compared to examine the implementation practicability.

A low value of MAE, RMSE, or MAPE indicates good performance; a short training time is desirable. SI is the mean of the sum of normalized values and indicates the overall model performance; it ranges from zero to one and zero indicates the best performance among all models.  Table 1 provides the formulas for the performance measures.

## 4. Analytical Results and Discussion

### 4.1. Experimental Settings

#### 4.1.1. Software and Hardware

Model building and testing were implemented in Anaconda software with the Python programming language on a machine (computer) with an NVIDIA GeForce RTX 2080 Ti graphics card. The Jupyter Notebook application in Anaconda [49] was used to display the inputs and outputs of the prediction models. Python packages support specific programming tasks and protect against their incompatibility. Numerous Python packages, which are available for use with Anaconda, are used (such as NumPy, pandas, and matplotlib). For building and testing the DNN models, the TensorFlow package [50] is used. For building and testing the CNN-based models, the Keras Application package [51] is used.

In particular, the Keras Application package supports the implementation of CNN models for prediction, fine-tuning, and feature extraction. It provides CNN models with pretrained weights from “ImageNet.” The package also provides a transfer learning feature that helps solve the practical problem of a lack of data resources and improves the accuracy of prediction using pretrained weights.  Table 2 presents information about the models, with accuracies that were obtained using the 2012 ILSVRC ImageNet validation set [51]. The depth refers to the number of layers in the Keras Applications' CNN model, including the activation layer, batch normalization layer, and other layers.

#### 4.1.2. Collection and Preprocessing of Data

A total of 8,310 data samples about ready-mixed concrete, relating to 32 variables, were collected from 2001 to 2019 by the Taiwan Construction Research Institute (TCRI). The data were split at the time of data sample collection to enable a prediction model to be built using historical data and tested using new data.

Accordingly, 339 data samples that covered one year (2019) were used in the testing process and the remaining 7,971 data samples were used in the training process. Of the 339 data samples for testing, 15 were removed because the value of compressive strength was missing, creating a test set of 324 data samples. The 7,971 data samples for training were further preprocessed according to the practical recommendations by a panel of domain experts in TCRI.

Among the 32 variables, the manufacturer's name, category of data, and date of collection were removed because the corresponding data were apparently irrelevant to the variability of concrete compressive strength. Ten other variables were removed because data were incomplete; these were the amount of admixture, the surface moisture content of sand (from a computer report and sieve analysis, respectively), silt charge, fineness modulus of sand, the strength of cement, specific surface area of cement, percentage of active blast furnace slag, fineness of blast furnace slag, and the ratio of water-reducing admixtures.

Totally, there are 19 concrete variables to be used for the prediction of the concrete compressive strength. One output variable is the test value of ready-mixed concrete compressive strength, and the other 18 input variables are the design strength of concrete, target strength of concrete, slump test, chloride ion content, temperature (temperature of the concrete taken on site), water-binder ratio, the water content of concrete, cementitious material consumption, cement ratio, amount of cement, amount of slag powder, amount of fly ash, amount of fine aggregate, amount of coarse aggregate, sand ratio, location (north), location (middle), and location (south).

The preprocessed data were processed again to yield three sets of data with different variables for use in numerical experiments for various purposes. Dataset 1 included 13 variables that are recommended by the TCRI; dataset 2 included 7 variables that are frequently used in prior studies [52–55] on the prediction of compressive strength; and dataset 3 included the resulting 18 variables after preprocessing. Tables 3–5 display the variables in the dataset, the number of data points in the datasets, and the descriptive statistics of variables in the datasets, respectively.

#### 4.1.3. Converting Numerical Data into Images

**Figure 20:**
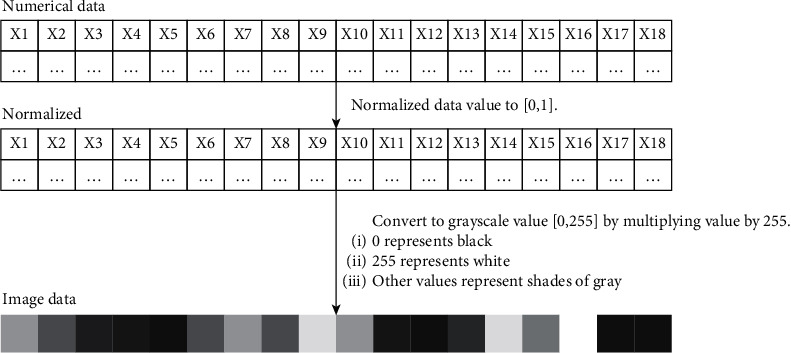
Conversion example of numerical data to image data.

The original numerical data were converted to images to be used as inputs to the CNN-based models. Each collection of values in a data sample for concrete was represented as an image. To create the image, the numerical data were first normalized to values between 0 and 1. These normalized data were then multiplied by 255 to encode them as grayscale values between 0 and 255 (Figure 20). Black represents 0 and white represents 255.

**Figure 21:**
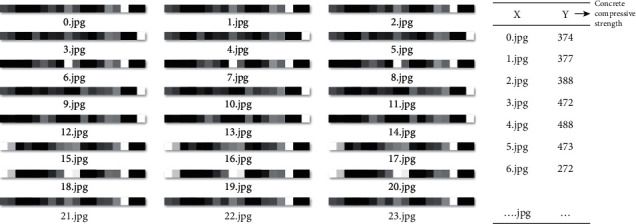
Input images and corresponding output labels.

For each of datasets 1 and 2, a total of 6705 images were created. For dataset 3, a total of 5856 images were created.  Figure 21 presents the example (dataset 3) of the labeling of the image data. Each image is labeled with the corresponding continuous output value, the compressive strength value of the ready-mixed concrete.

### 4.2. Implementation and Comparison

Prediction models and sensitivity experiments with various purposes were carried out (Table 6). Baseline models were used with the hyperparameters set to default values in the TensorFlow and Keras Applications. In the DNN, numerical data are input, while for the CNN-based models, the input numerical data are converted to image data. In this study, the size of the image input to each CNN-based model was the minimum possible size to meet the practical needs.

#### 4.2.1. Deep Learning Models and Performance

Since the same model and hyperparameters yielded different model performance values in different runs, each model was tested five times and the average model performance value was taken as the actual. For both the CNN and DNN models, the loss function was set to be the MSE. In the DNN model, 50 hidden layers with selected numbers of hidden nodes (Table 7) had the best prediction accuracy in comparison with other numbers of hidden layers and other numbers of hidden nodes. The architecture was thus used to build the baseline DNN prediction model.

Tables 8–10 compare the performances of the DL models in predicting the compressive strength of ready-mixed concrete when they are trained and tested using the given data. The results indicate that the CNN models, ResNet50V2, MobileNet, and DenseNet121, with their default parameters, all performed best on the three datasets, respectively. The CNN models, ResNet50V2, MobileNet, and DenseNet121, with image data, outperformed the baseline DNN model with numerical data. The results also indicate that the best CNN models on each dataset outperformed the DNN in terms of each performance metric, except for the training time.

#### 4.2.2. Optimized Convolutional Neural Network-Based Models

As CNN models, ResNet50V2, MobileNet, and DenseNet121, performed best in the corresponding datasets, a metaheuristic optimization algorithm, the jellyfish search (JS) optimizer, was used to optimize them. The CNN models were optimized to minimize the errors of prediction of the compressive strength of ready-mixed concrete using the best values of the hyperparameters.

The JS optimizer was used to find the best hyperparameter values in a set of ranges. Several hyperparameters of a CNN, such as the epsilon of batch normalization, batch size, epoch, learning rate, and dropout rate, were selected to be adjusted during the search herein. For DenseNet121, two additional hyperparameters were optimized—the growth rate and the reduction value.  Table 11 presents the default values of hyperparameters in the reference papers [34, 41] and the range of hyperparameters to be finetuned in this study.

Table 12 compares the performances of best CNN models using default hyperparameters and optimized by JS in predicting the compressive strength of ready-mixed concrete. The results indicate that using the JS optimizer on the hyperparameters improved the accuracy of the prediction models.  Table 13 shows the best hyperparameter settings for each optimized CNN model.

### 4.3. Influence of Feature and Image Pixel Orientation on Modeling Accuracy

To examine the sensitivity of the generalization ability of a prediction model, the resulting 18 variables (features) were experimented using the best CNN model (DenseNet121) by removing one of the variables and using the remaining variables for model training. For the location variables, three variables were removed simultaneously and the remaining variables were used for sensitivity analysis. These tests were conducted to investigate the effect of each feature (attribute) on the generalization ability of model prediction.  Table 14 displays the performance results with MAPEs, in which the lower value of the MAPE stands for the better model performance without the specified attribute. The experiment demonstrated that the MAPEs do not differ much from one another. However, the slight increase of MAPE in each numerical experiment comparing to the baseline MAPE (11.72%) implies the inclusion of those variables (*X*1–*X*3, *X*5, and *X*16–*X*18) has a positive impact on the prediction accuracy of ready-mixed concrete compressive strength.

Another numerical experiment was conducted to examine the influence of image pixel orientation (pixel row order) on the computer vision-based modeling performance. Two types of image pixel orientation (IPO) formed by the input attributes (pixels) were tested, namely, the original pixel array and the correlated pixel array, according to the correlation values between the input attributes and the compressive strength. Specifically, the input image data were shaped by arranging the input attributes (pixels) in random order and descending the pixels order by their correlation coefficients, respectively.

Table 15 displays the correlation coefficients between the input variables and the compressive strength of ready-mixed concrete. Ordering the IPO based on the magnitude of the correlation coefficients, two new datasets were created. One IPO is arranged by descending the original values of the correlation coefficients and the other IPO is arranged by descending their absolute values.

Table 16 presents the sensitivity analysis of image pixel orientation on the computer vision-based modeling performance. It is observable that all metrics with the correlated order of image pixel orientation show worse performance than that obtained using the original ordered image by the same optimized CNN model (JS-DenseNet121). Therefore, the analytical results indicate that the correlated order of image pixel orientation for the image converting of ready-mixed concrete data does not significantly influence the performance of the prediction model.

## 5. Conclusions

The effectiveness of computer vision in predicting the compressive strength of ready-mixed concrete was analyzed to improve the predictions thereof. Deep learning (DL) models were constructed by imaging the numerical data as inputs to predict the compressive strength of ready-mixed concrete. Various prediction models were compared and the best DL prediction models were identified for different sets of input concrete-related features and optimized after their performances were further analyzed.

The models for the prediction of concrete compressive strength are frequently built with the use of cross-validation or random split in-sample data for evaluating prediction accuracy, which often gives optimistic results (overfitting) in the training/test process while exhibiting poor performance in future use. It's mainly because the processes, materials, machines, and technicians that are involved to manufacture ready-mixed concrete in batch plants are being continually improved and replaced periodically. Up-to-date samples for ready-mixed concrete might be derived differently from the evolving development of batch processes.

A prediction model is built using historical data; it uses newly collected data, which should be irrelevant to the training data samples, to make predictions; therefore, the optimality of using random split in-sample data to test models in the prediction of concrete compressive strength in the literature is now doubted. To capture the actual performance of predicting the compressive strength of concrete, out-of-sample data (newly collected data) should be used for model testing to avoid potential information leakage. Although the model accuracy may be decreased in comparison with that obtained by in-sample cross-validation or randomly split data for training and test, using such an approach for the out-of-sample test reflects the real predictive performance in practice.

Furthermore, CNN-oriented models are often trained without tuning the hyperparameters. This study adopts a metaheuristic optimization algorithm to optimize the prediction model. The predictive accuracy of computer vision-based deep learning models was improved herein using the jellyfish search (JS) metaheuristic optimization algorithm. The JS optimizer finds the best hyperparameters, optimizing the performance metrics of the CNN models. This study contributes to the novel application of the computer vision-based method, which integrates the latest CNN models with a newly developed JS optimizer to predict the compressive strength of ready-mixed concrete. The analytical experiments show that modeling with image-converting data outperforms the models using the original numerical data.

In this investigation, the training data were samples on ready-mixed concrete only. Using data on high-performance concrete or more complex engineering data would improve this work of the computer vision approach to predicting a numerical output like the compressive strength of concrete. More cases should be studied to confirm the effectiveness of imaging data on ready-mixed concrete and other types of concrete to identify patterns of compressive strength by the bio-inspired metaheuristic optimization of computer vision-based deep learning models.

Future studies could consider environment-oriented factors that may affect the ready-mixed concrete compressive strength, such as the type of manufacturing equipment, transporting process of concrete, and the handling speed of on-site operators in addition to the material-oriented attributes herein. A fair comparison between laboratory-determined concrete compressive strength and on-site evaluation of concrete compressive strength should be investigated.

## Figures and Tables

**Table 1:** Performance metrics.

Performance metric	Formula
Mean absolute error (MAE)	1/*n*∑_*i*=1_^*n*^|*y* − *y*′|
Mean squared error (MSE)	1/*n*∑_*i*=1_^*n*^(*y*′ − *y*)^2^
Root mean squared error (RMSE)	(1/*n*∑_*i*=1_^*n*^(*y*′ − *y*)^2^)^1/2^
Mean absolute percentage error (MAPE)	1/*n*∑_*i*=1_^*n*^|(*y* − *y*′)/*y*|
Synthesis index (SI)	1/*m*∑_*i*=1_^*m*^|(*P* − *P*_min_)/(*P*_max_ − *P*_min_)|

*Note*. *n*, number of predictions; *y*, actual value; *y*′, predicted value; *m*, number of performance metrics; *P*, value of the performance metric; *P*_min_, minimum value of performance metric; *P*_max_, maximum value of performance metric.

**Table 2:** Convolutional neural network-based models in the Keras Application.

Model	Top 1 accuracy	Top 5 accuracy	Depth	Size (MB)	Parameters	Reference

VGG16	0.713	0.901	23	528	138,357,544	[33]
VGG19	0.713	0.900	26	549	143,667,240	
ResNet50	0.749	0.921	—	98	25,636,712	[34]
ResNet101	0.764	0.928	—	171	44,707,176	
ResNet152	0.766	0.931	—	232	60,419,944	
ResNet50V2	0.760	0.930	—	98	25,613,800	[35]
ResNet101V2	0.772	0.938	—	171	44,675,560	
ResNet152V2	0.780	0.942	—	232	60,380,648	
InceptionV3	0.779	0.937	159	92	23,851,784	[36]
InceptionResNetV2	0.803	0.953	572	215	55,873,736	[37]
Xception	0.790	0.945	126	88	22,910,480	[38]
MobileNet	0.704	0.895	88	16	4,253,864	[39]
MobileNetV2	0.713	0.901	88	14	3,538,984	[40]
DenseNet121	0.750	0.923	121	33	8,062,504	[41]
DenseNet169	0.762	0.932	169	57	14,307,880	
DenseNet201	0.773	0.936	201	80	20,242,984	
NASNetMobile	0.744	0.919	—	23	5,326,716	[42]
NASNetLarge	0.825	0.960	—	343	88,949,818	
EfficientNetB0	—	—	—	29	5,330,571	[43]
EfficientNetB1	—	—	—	31	7,856,239	
EfficientNetB2	—	—	—	36	9,177,569	
EfficientNetB3	—	—	—	48	12,320,535	
EfficientNetB4	—	—	—	75	19,466,823	
EfficientNetB5	—	—	—	118	30,562,527	
EfficientNetB6	—	—	—	166	43,265,143	
EfficientNetB7	—	—	—	256	66,658,687	

**Table 3:** Variables in the datasets.

Dataset variable	Dataset 1	Dataset 2	Dataset 3

Design strength of concrete	—	—	✓
Target strength of concrete	—	—	✓
Slump test	—	—	✓
Chloride ion content	—	—	✓
Temperature	—	—	✓
Water-binder ratio	✓	✓	✓
Water content of concrete	✓	✓	✓
Cementitious material consumption	✓	—	✓
Cement ratio	✓	—	✓
Amount of cement	✓	✓	✓
Amount of slag powder	✓	✓	✓
Amount of fly ash	✓	✓	✓
Amount of fine aggregate	✓	✓	✓
Amount of coarse aggregate	✓	✓	✓
Sand ratio	✓	—	✓
Location (north)	✓	—	✓
Location (middle)	✓	—	✓
Location (south)	✓	—	✓
Compressive strength test	✓	✓	✓

*Note*. Dataset 1 = industry recommendation; dataset 2 = suggested by research community; dataset 3 = all features considered. Variables in dataset 2 are frequently used to determine the compressive strength of concrete in the literature.

**Table 4:** Number of data points in the datasets.

Number of data points	Dataset 1	Dataset 2	Dataset 3
Number of total samples	6705	6705	5856
Number of training samples	6381	6381	5532
Number of testing samples	324	324	324
Number of input variables	13	7	18
Number of output variables	1	1	1

**Table 5:** Descriptive statistics of variables from the datasets.

Variables		Unit	Minimum	Maximum	Average

*Dataset 1—industry recommendation*					
*X*6	Water-binder ratio	—	0.25	0.87	0.52
*X*7	Water content of concrete	(kg/m^3^)	121.00	250.25	184.98
*X*8	Cementitious material consumption	(kg/m^3^)	209.00	690.00	361.15
*X*9	Cement ratio	(%)	30.67	100.00	70.33
*X*10	Amount of cement	(kg/m^3^)	99.20	507.00	255.43
*X*11	Amount of slag powder	(kg/m^3^)	0.00	209.35	68.90
*X*12	Amount of fly ash	(kg/m^3^)	0.00	180.00	36.82
*X*13	Amount of coarse aggregate	(kg/m^3^)	344.24	1281.00	919.30
*X*14	Amount of fine aggregate	(kg/m^3^)	468.00	1376.96	860.22
*X*15	Sand ratio	(%)	0.00	80.00	48.32
*X*16	Location (north)	—	0.00	1.00	0.44
*X*17	Location (middle)	—	0.00	1.00	0.12
*X*18	Location (south)	—	0.00	1.00	0.44
*Y*	Compressive strength test	(kgf/cm^2^)	125.00	724.00	343.49

*Dataset 2—suggested by the research community*					
*X*6	Water-binder ratio	—	0.25	0.87	0.52
*X*7	Water content of concrete	(kg/m^3^)	121.00	250.25	184.98
*X*10	Amount of cement	(kg/m^3^)	99.20	507.00	255.43
*X*11	Amount of slag powder	(kg/m^3^)	0.00	209.35	68.90
*X*12	Amount of fly ash	(kg/m^3^)	0.00	180.00	36.82
*X*13	Amount of fine aggregate	(kg/m^3^)	468.00	1376.96	860.22
*X*14	Amount of coarse aggregate	(kg/m^3^)	344.24	1281.00	919.30
*Y*	Compressive strength test	(kgf/cm^2^)	125.00	724.00	343.49

*Dataset 3—all features considered*					
*X*1	Design strength of concrete	(kgf/cm^2^)	140.00	420.00	254.40
*X*2	Target strength of concrete	(kgf/cm^2^)	160.00	660.00	320.27
*X*3	Slump test	(cm)	8.50	69.00	19.48
*X*4	Chloride ion content	(%)	0.00	0.14	0.04
*X*5	Temperature	(°C)	14.00	35.00	26.19
*X*6	Water-binder ratio	—	0.25	0.83	0.52
*X*7	Water content of concrete	(kg/m^3^)	121.00	250.25	184.87
*X*8	Cementitious material consumption	(kg/m^3^)	209.00	690.00	363.42
*X*9	Cement ratio	(%)	30.67	100.00	70.10
*X*10	Amount of cement	(kg/m^3^)	99.20	507.00	256.17
*X*11	Amount of slag powder	(kg/m^3^)	0.00	209.35	68.85
*X*12	Amount of fly ash	(kg/m^3^)	0.00	180.00	38.40
*X*13	Amount of fine aggregate	(kg/m^3^)	468.00	1376.96	860.88
*X*14	Amount of coarse aggregate	(kg/m^3^)	344.24	1281.00	916.43
*X*15	Sand ratio	(%)	0.00	80.00	48.41
*X*16	Location (north)	—	0.00	1.00	0.48
*X*17	Location (middle)	—	0.00	1.00	0.13
*X*18	Location (south)	-	0.00	1.00	0.39
*Y*	Compressive strength	(kgf/cm^2^)	162.00	724.00	344.82

**Table 6:** Experimental settings.

Research task	Data type	Purpose	Method
Comparison of deep learning models	Numerical data and image data	Search for the best CNN model (using image data) and compare the best CNN model with a DNN model (using numerical data)	CNNs and DNN: VGG, ResNet, ResNetV2, InceptionV3, InceptionResNetV2, MobileNets, MobileNetV2, NASNet, EfficientNets, DenseNet
Construction of optimized deep learning models	Image data	Enhance the best-performing models with optimized hyperparameters	Optimizing deep learning models by jellyfish search algorithm

**Table 7:** Number of hidden nodes in each hidden layer of the deep neural network (DNN).

Hidden layer	1^st^	2^nd^	3^rd^	4^th^	5^th^	6^th^	7^th^	8^th^–10^th^	11^th^–20^th^	21^st^–30^th^	31^st^–40^th^	41^st^–50^th^
Number of hidden nodes	4096	2048	1024	512	256	128	64	32	16	8	4	2

**Table 8:** Deep learning model performance on dataset 1.

Model	Training time (h:m:s)	MAPE (%)	RMSE (kgf/cm^2^)	MAE (kgf/cm^2^)	SI

Xception	2:31:01	14.0264	76.4217	57.7471	0.285
VGG16	0:17:58	15.9598	85.6755	65.6588	0.249
VGG19	0:21:16	14.8719	79.9609	61.1835	0.147
ResNet50	0:18:28	15.0252	80.8499	62.5910	0.164
ResNet101	0:32:30	14.3817	75.4145	57.5325	0.091 (3)
ResNet152	0:44:12	14.3462	78.5345	59.2920	0.144
ResNet50V2	0:16:56	13.8000	73.7818	56.4419	0.027 (1)
ResNet101V2	0:29:17	14.7393	74.4318	58.7149	0.100
ResNet152V2	0:43:11	16.2188	85.7025	67.9747	0.318
InceptionV3	0:45:14	14.2727	75.2054	58.0613	0.111
InceptionResNetV2	1:43:38	14.7849	77.9702	60.6185	0.264
MobileNet	0:10:35	15.1504	79.4783	62.0158	0.141
MobileNetV2	0:12:09	17.2442	82.0049	63.9462	0.244
DenseNet121	0:18:49	15.6411	82.3700	64.8698	0.213
DenseNet169	0:24:41	15.3712	80.3540	63.4890	0.190
DenseNet201	0:31:28	15.4292	80.7504	63.7175	0.207
NASNetMobile	0:35:50	15.8436	82.1353	64.5839	0.244
EfficientnetB0	0:18:10	14.2585	75.3426	58.3330	0.069 (2)
EfficientnetB1	0:27:30	14.9977	80.3189	61.8780	0.169
EfficientnetB2	0:28:38	15.4503	80.6015	63.4244	0.200
EfficientnetB3	0:35:30	14.9118	80.7490	61.6706	0.181
EfficientnetB4	0:45:16	14.6289	79.1150	61.1313	0.173
EfficientnetB5	0:59:30	14.5750	76.2138	59.6796	0.164
EfficientnetB6	1:13:08	15.0979	81.5700	62.4580	0.261
EfficientnetB7	1:42:25	14.4783	74.6023	59.1117	0.218
DNN	0:00:46	21.4910	112.2759	87.9198	0.750

**Table 9:** Deep learning model performance on dataset 2.

Model	Training time (h:m:s)	MAPE (%)	RMSE (kgf/cm^2^)	MAE (kgf/cm^2^)	SI

Xception	1:12:19	17.2430	89.4229	70.1275	0.493
VGG16	0:10:26	18.3202	100.0902	73.6012	0.387
VGG19	0:12:32	17.7289	98.4250	74.1325	0.373
ResNet50	0:12:24	15.0208	78.1490	61.1607	0.105
ResNet101	0:21:34	14.5379	74.5514	58.2454	0.086 (2)
ResNet152	0:30:17	16.5355	90.8698	67.9069	0.321
ResNet50V2	0:11:28	15.7710	80.7428	63.8803	0.154
ResNet101V2	0:20:19	15.1000	76.9871	60.4406	0.124
ResNet152V2	0:28:43	14.4184	73.6713	57.4732	0.098
InceptionV3	0:27:04	16.4996	87.7770	69.3648	0.302
InceptionResNetV2	1:02:49	15.8074	84.5667	66.3267	0.371
MobileNet	0:06:18	15.0699	77.6579	61.3514	0.084 (1)
MobileNetV2	0:07:26	18.6955	83.8692	68.5122	0.264
DenseNet121	0:12:41	15.3784	83.6683	64.4323	0.167
DenseNet169	0:17:17	15.5968	85.7416	65.6683	0.209
DenseNet201	0:22:02	14.9204	83.5047	62.5952	0.175
NASNetMobile	0:20:32	22.3714	115.6740	93.5611	0.745
EfficientnetB0	0:12:27	15.0674	80.2897	61.9535	0.124
EfficientnetB1	0:17:52	15.5417	81.8874	63.5534	0.174
EfficientnetB2	0:18:12	14.5659	77.0040	59.4023	0.096 (3)
EfficientnetB3	0:21:51	15.4034	81.4909	63.2014	0.180
EfficientnetB4	0:27:17	15.7882	83.7080	64.3034	0.228
EfficientnetB5	0:36:30	14.9359	78.5506	60.4780	0.185
EfficientnetB6	0:45:47	15.0286	78.3096	61.4788	0.225
EfficientnetB7	1:01:55	15.3541	80.7350	62.9946	0.313
DNN	0:00:40	23.9215	119.4923	95.5619	0.750

**Table 10:** Deep learning model performance on dataset 3.

Model	Training time (h:m:s)	MAPE (%)	RMSE (kgf/cm^2^)	MAE (kgf/cm^2^)	SI

Xception	2:59:21	13.0524	64.3952	51.9907	0.326
VGG16	0:20:24	13.8902	69.6804	56.1444	0.165
VGG19	0:23:28	14.1965	69.1497	55.3852	0.169
ResNet50	0:20:26	13.9585	70.9084	57.2012	0.178
ResNet101	0:34:28	11.6479	59.5678	47.1273	0.049 (2)
ResNet152	0:48:51	11.7348	60.4651	47.2528	0.076
ResNet50V2	0:18:31	12.3769	64.2470	51.1966	0.083
ResNet101V2	0:31:55	12.1501	63.4072	49.7640	0.086
ResNet152V2	0:46:54	12.0281	61.4381	48.0813	0.087
InceptionV3	0:51:37	13.3665	64.9479	52.1857	0.157
InceptionResNetV2	1:57:42	13.5490	64.0620	51.9634	0.248
MobileNet	0:12:27	13.1265	65.2462	52.4753	0.100
MobileNetV2	0:14:00	13.0155	60.0301	47.8787	0.053
DenseNet121	0:20:09	11.7167	59.3511	47.1034	0.029 (1)
DenseNet169	0:25:51	11.7929	61.0411	47.9452	0.051 (3)
DenseNet201	0:32:50	11.6047	60.6139	47.7109	0.054
NASNetMobile	0:41:08	24.6483	109.9440	93.7760	0.780
EfficientnetB0	0:20:37	12.9179	64.7033	52.1055	0.103
EfficientnetB1	0:30:28	13.3862	68.6528	55.0783	0.159
EfficientnetB2	0:31:25	13.4363	68.1339	54.9089	0.159
EfficientnetB3	0:38:59	13.2016	67.0390	53.1422	0.150
EfficientnetB4	0:49:39	13.1075	65.5639	52.3979	0.153
EfficientnetB5	1:09:29	13.2787	67.5948	54.1713	0.203
EfficientnetB6	1:26:14	12.4606	63.7319	50.5952	0.174
EfficientnetB7	1:55:15	12.8867	63.4471	51.0061	0.224
DNN	0:00:44	22.8024	116.0370	90.6988	0.698

**Table 11:** Hyperparameter settings for deep learning models.

Hyperparameter	Literature value	Search range in this study

*ResNet50V2* [34]		
Batch normalization-epsilon	1.001*e* − 5	[1.001*e* − 5, 0.00005, 0.0001, 0.0005, 0.001]
Batch size	64, 256	[8, 16, 32, 64]
Epochs	40, 90, 300	[10, 20, 30, 40, 50, 60, 70, 80, 90, 100]
ADAM-learning rate	0.1	[0.001, 0.005, 0.01, 0.05, 0.1]
Dropout rate	0.5	0.00–0.99

*MobileNet* [39]		
Batch size	64, 256	[8, 16, 32, 64]
Epochs	40, 90, 300	[10, 20, 30, 40, 50, 60, 70, 80, 90, 100]
ADAM-learning rate	0.1	[0.001, 0.005, 0.01, 0.05, 0.1]
Dropout rate	0.5	0.00–0.99

*DenseNet121* [41]		
Growth rate	32	12–48
Batch normalization-epsilon	1.001*e* − 5	[1.001*e* − 5, 0.00005, 0.0001, 0.0005, 0.001]
Batch size	64, 256	[8, 16, 32, 64]
Epochs	40, 90, 300	[10, 20, 30, 40, 50, 60, 70, 80, 90, 100]
Reduction	0.5	0.1–1.0
ADAM-learning rate	0.1	[0.001, 0.005, 0.01, 0.05, 0.1]
Dropout rate	0.2	0.00–0.99

**Table 12:** Performance of the best and optimized CNN models.

Dataset	Model	MAPE (%)	RMSE (kgf/cm^2^)	MAE (kgf/cm^2^)
1	ResNet50V2	13.8000	73.7818	56.4419
	JS-ResNet50V2	13.1327	68.5794	52.4591

2	MobileNet	17.0406	91.6945	71.1198
	JS-MobileNet	17.0671	90.4711	70.0870

3	DenseNet121	11.7167	59.3511	47.1034
	JS-DenseNet121	11.5443	58.4346	45.8917

**Table 13:** Optimal hyperparameters of the best CNN models.

Hyperparameter	Optimal value
*JS-ResNet50V2*	
Batch normalization-epsilon	0.0005
Batch size	64
Epochs	100
ADAM-learning rate	0.001
Dropout rate	0.26

*JS-MobileNet*	
Batch size	16
Epochs	70
ADAM-learning rate	0.001
Dropout rate	0.65

*JS-DenseNet121*	
Growth rate	38
Batch normalization-epsilon	0.00005
Batch size	64
Epochs	90
Reduction	0.7
ADAM-learning rate	0.001
Dropout rate	0.33

**Table 14:** Sensitivity analysis of input features.

No.	*X*1	*X*2	*X*3	*X*4	*X*5	*X*6	*X*7	*X*8	*X*9	*X*10	*X*11	*X*12	*X*13	*X*14	*X*15	*X*16	*X*17	*X*18	MAPE (%)

1	—	✓	✓	✓	✓	✓	✓	✓	✓	✓	✓	✓	✓	✓	✓	✓	✓	✓	11.94
2	✓	—	✓	✓	✓	✓	✓	✓	✓	✓	✓	✓	✓	✓	✓	✓	✓	✓	11.91
3	✓	✓	—	✓	✓	✓	✓	✓	✓	✓	✓	✓	✓	✓	✓	✓	✓	✓	11.95
4	✓	✓	✓	—	✓	✓	✓	✓	✓	✓	✓	✓	✓	✓	✓	✓	✓	✓	11.66
5	✓	✓	✓	✓	—	✓	✓	✓	✓	✓	✓	✓	✓	✓	✓	✓	✓	✓	11.99
6	✓	✓	✓	✓	✓	—	✓	✓	✓	✓	✓	✓	✓	✓	✓	✓	✓	✓	11.01
7	✓	✓	✓	✓	✓	✓	—	✓	✓	✓	✓	✓	✓	✓	✓	✓	✓	✓	11.32
8	✓	✓	✓	✓	✓	✓	✓	—	✓	✓	✓	✓	✓	✓	✓	✓	✓	✓	11.05
9	✓	✓	✓	✓	✓	✓	✓	✓	—	✓	✓	✓	✓	✓	✓	✓	✓	✓	11.57
10	✓	✓	✓	✓	✓	✓	✓	✓	✓	—	✓	✓	✓	✓	✓	✓	✓	✓	11.17
11	✓	✓	✓	✓	✓	✓	✓	✓	✓	✓	—	✓	✓	✓	✓	✓	✓	✓	11.63
12	✓	✓	✓	✓	✓	✓	✓	✓	✓	✓	✓	—	✓	✓	✓	✓	✓	✓	11.69
13	✓	✓	✓	✓	✓	✓	✓	✓	✓	✓	✓	✓	—	✓	✓	✓	✓	✓	11.29
14	✓	✓	✓	✓	✓	✓	✓	✓	✓	✓	✓	✓	✓	—	✓	✓	✓	✓	11.59
15	✓	✓	✓	✓	✓	✓	✓	✓	✓	✓	✓	✓	✓	✓	—	✓	✓	✓	11.26
16	✓	✓	✓	✓	✓	✓	✓	✓	✓	✓	✓	✓	✓	✓	✓	—	—	—	11.98
17	✓	✓	✓	✓	✓	✓	✓	✓	✓	✓	✓	✓	✓	✓	✓	✓	✓	✓	11.72

*Note. X*1 = design strength of concrete, *X*2 = target strength of concrete, *X*3 = slump test, *X*4 = chloride ion content, *X*5 = temperature, *X*6 = water-binder ratio, *X*7 = water content of concrete, *X*8 = cementitious material consumption, *X*9 = cement ratio, *X*10 = amount of cement, *X*11 = amount of slag powder, *X*12 = amount of fly ash, *X*13 = amount of fine aggregate, *X*14 = amount of coarse aggregate, *X*15 = sand ratio, *X*16 = location (north), *X*17 = location (middle), and *X*18 = location (south).

**Table 15:** Correlation between the feature and compressive strength of ready-mixed concrete.

Feature	Correlation coefficient between feature and *Y*
*X*1	0.75
*X*2	0.82
*X*3	0.23
*X*4	0.05
*X*5	−0.15
*X*6	−0.74
*X*7	−0.15
*X*8	0.73
*X*9	0.14
*X*10	0.46
*X*11	0.05
*X*12	0.02
*X*13	−0.44
*X*14	−0.06
*X*15	−0.25
*X*16	0.24
*X*17	0.06
*X*18	−0.29

*Note. X*1 = design strength of concrete, *X*2 = target strength of concrete, *X*3 = slump test, *X*4 = chloride ion content, *X*5 = temperature, *X*6 = water-binder ratio, *X*7 = water content of concrete, *X*8 = cementitious material consumption, *X*9 = cement ratio, *X*10 = amount of cement, *X*11 = amount of slag powder, *X*12 = amount of fly ash, *X*13 = amount of fine aggregate, *X*14 = amount of coarse aggregate, *X*15 = sand ratio, *X*16 = location (north), *X*17 = location (middle), *X*18 = location (south), and *Y* = compressive strength of ready-mixed concrete.

**Table 16:** Results of the order importance analysis of the image-like dataset.

Image pixel orientation	Type of pixel order	MAPE (%)	RMSE (kgf/cm^2^)	MAE (kgf/cm^2^)
Original order	Random arrangement	11.5443	58.4346	45.8917
Correlated order	Descending by correlated values	12.0831	61.3435	48.1922
	Descending by absolute correlated values	12.5888	64.5037	50.7435

## Data Availability

The datasets, codes, and replication of results generated and/or analyzed during the current study are available from the corresponding author on reasonable request.

## Abbreviations

AI: Artificial intelligence
ANN: Artificial neural network
BN: Batch normalization
CNN/ConvNet: Convolutional neural network
DenseNet: Dense convolutional network
DL: Deep learning
DNN: Deep neural network
FNN: Feedforward neural network
IN: Instance normalization
IPO: Image pixel orientation
JS: Jellyfish search
LN: Layer normalization
MAE: Mean absolute error
MAPE: Mean absolute percentage error
MSE: Mean squared error
NASNet: Neural architecture search network
ReLU: Rectified linear unit
ResNet: Residual neural network
RF: Random forest
RMSE: Root mean squared error
RNN: Recurrent neural network
SI: Synthesis index
SVR: Support vector regression
TCRI: Taiwan Construction Research Institute
VGG: Visual geometry group
Xception: Extreme inception.

## Symbols

*w* × *h* × *c*: Width of image × height of image × number of channels
*m*: Dimension of an input image
*n*: Filter size
*C*: Convolution map
*I*: Input image data
Θ: Convolution operation
*F*: Filter
*o*: Dimension of convolution map
*s*: Stride
zp: Zero padding
*f*: Nonlinear activation function
*C*_*m*_: Convolution map after applying the nonlinear activation function *f*
*P*_*m*_: Pooling map
*P*_*o*_: Pooling operation
*Y* ^*i*^: Model output of the ith fully connected hidden layer
*H* ^*i*^: Weight sum vector
*B* ^*i*^: The activation level of the artificial neurons
*BN*(*x*): Batch normalization at a given layer from x
*γ*: Scale parameter for the channel
*β*: Shift parameter for the channel
*μ*_*B*_: Mean of the batch
*σ*_*B*_: Standard deviation of the batch
∗: Element-wise product
*f* ^*l*^(*x*_*i*_): Original feature
${\tilde{f}}^{\;l}\left(x_{i}\right)$: Distorted features
*m*_*i*_^*l*^: Binary mask
d^l^: Dimension of the feature map of the l-th layer
$\overset{⟶}{\text{trend}}$: Direction of the ocean current
*X* ^*∗*^: Jellyfish with the optimal location
*μ*: Average location of all jellyfish
*X*_*i*_: Jellyfish of interest
*t*: Time specified as an iteration number
*X*_*i*_(*t*): Current location of a jellyfish
*X*_*i*_(*t*+1): New location of a jellyfish after a movement
rand(0,1): Random number between 0 and 1
*Ub*: Upper bounds of the search spaces
*Lb*: Lower bounds of the search spaces
*X*_*j*_: Jellyfish other than the jellyfish of interest
*f*(*X*_*i*_): Quantity of food at the location of *X*_*i*_
*f*(*X*_*j*_): Quantity of food at the location of *X*_*j*_
$\overset{⟶}{\text{Direction}}$: Direction of active motion (type B) of jellyfish
*c*(*t*): Time control function
Max_iter_: Maximum number of iterations
*n*: Number of predictions
*y*: Actual value
*y*′: Predicted value
*m*: Number of performance metrics
*P*: Value of performance metric
*P*_min_: Minimum value of performance metric
*P*_max_: Maximum value of performance metric.
